# Silver Nanoparticles and Antibiotics: A Promising Synergistic Approach to Multidrug-Resistant Infections

**DOI:** 10.3390/microorganisms13040952

**Published:** 2025-04-21

**Authors:** Eudald Casals, Muriel F. Gusta, Neus Bastus, Jordi Rello, Victor Puntes

**Affiliations:** 1 Vall d’Hebron Institut de Recerca (VHIR), 08035 Barcelona, Spain; eudaldcm@gmail.com; 2 Premium Research SL, 19003 Guadalajara, Spain; 3 Catalan Institute of Nanoscience & Nanotechnology (ICN2), Consejo Superior de Investigaciones Científicas (CSIC), The Barcelona Institute of Science and Technology (BIST), Campus UAB, 08193 Bellaterra, Spain; muriel.freixanet@icn2.cat (M.F.G.); neus.bastus@icn2.cat (N.B.); 4 Networking Research Centre for Bioengineering Biomaterials, and Nanomedicine (CIBER-BBN), 28029 Madrid, Spain; 5 CIBER de Enfermedades Respiratorias (CIBERES), Instituto de Salud Carlos III, 28029 Madrid, Spain; 6 Formation, Recherche, Evaluation (FOREVA) Research Unit, CHU Nîmes, 30029 Nîmes, France; 7 Institució Catalana de Recerca i Estudis Avançats (ICREA), 08010 Barcelona, Spain

**Keywords:** antibiotic resistance, silver nanoparticles, multidrug-resistant pathogens, antimicrobial synergy, controlled ion release

## Abstract

The escalating threat of antibiotic resistance demands innovative strategies against multidrug-resistant (MDR) microorganisms, particularly in hospital settings where such infections represent a major global health challenge. Since the rapid growth of nanotechnology interdisciplinary research and funding programs in the 2000s, silver ions have re-emerged as potent antimicrobial agents, offering a promising complement to conventional therapies. This therapeutic potential is nowadays explored through the use of silver nanoparticles (AgNPs) as sources for silver ions release. Recent studies have shown that controlled silver ion release enhances the efficacy of common antibiotics. This can be attributed to the energetically demanding nature of the bacterial response to silver, which weakens bacterial metabolism and, in turn, overwhelms bacterial defenses and increases antibiotic effectiveness. Herein, historical insights into the use of colloidal silver and AgNPs are combined with a review of recent research on the exploitation of the synergistic effect between AgNPs and antibiotics as a promising strategy against MDR pathogens.

## 1. Introduction

In his 1945 Nobel lecture, Alexander Fleming already sounded a cautionary note about the potential pitfalls of antibiotic therapy due to misuse and overuse [1]. Fast forward seventy years, untreatable infections have become an unsettling and unfortunate reality, especially hospital-acquired infections in clinical settings [2]. In hospitals, indirect transmission of pathogens represents a major clinical threat all over the world, in particular with the recent emergence of pathogens resistant to most or all available antimicrobial drugs [3]. This presents a considerable burden in terms of morbidity, mortality, and healthcare-associated costs [4,5,6,7,8]. The reasons behind the escalating resistance are multifaceted, including the overuse or improper use of antibiotics and the widespread use in industrial farming and the food industry [4,9,10]. Driven by this threat of antibiotic resistance, regulatory agencies, pharmaceutical industries, investors, and basic and clinical researchers are devoting resources and efforts to provide effective antibiotics for infections caused by multidrug-resistant (MDR) microorganisms. Examples include the use of monoclonal antibodies, mesenchymal stem cells, anti-virulence agents, or bacteriophages [11,12,13,14].

In this scenario, the antimicrobial properties of silver ions, known for a long time, have re-emerged as a topic of interest, especially with the rise of nanotechnology in the 2000s [15]. Since then, research on the potential of silver nanoparticles (AgNPs) as silver ion reservoirs has been continuously growing [16]. AgNPs are non-bioactive but slowly dissolve in aqueous solutions and physiological conditions, releasing silver ions that exert biological activity [17,18]. This allows for precise and sustained controlled release through their gradual oxidation and posterior corrosion, providing long-term protection even at minimal NP concentrations [19]. In this sense, AgNPs behave in analogy to a drug delivery system, in which the particle contains a concentrated amount of an active species, the silver ion, which is transported to and released near biological target sites. NP size, shape, crystal structure, and surface state can be adjusted with precision to determine the oxidation and dissolution (delivery) rate [20].

Silver exhibits a pleiotropic mode of action, inducing changes in bacterial cells that can increase and potentially restore susceptibility to antibiotics [21,22,23,24]. Silver ions and AgNPs can interact with the phospholipid bilayer of bacterial cell membranes, increasing membrane permeability and causing leakage of cellular contents [25,26]. This, in turn, facilitates antibiotic internalization, ultimately leading to cell death [17]. Silver ions and AgNPs also generate reactive oxygen species (ROS) via Fenton-like reactions, inducing oxidative damage to proteins, lipids, and DNA [27,28,29,30,31,32]. Additionally, the strong affinity of silver ions for sulfur results in irreversible Ag-S bonds and Ag_2_S precipitates, which interfere with critical cellular processes, including disulfide bond formation, sulfidation, and iron homeostasis [33]. Moreover, silver ions target thiol-dependent enzymes like thioredoxin and glutathione, a promising bactericidal approach against multidrug-resistant (MDR) bacteria [34]. They also interact with bacterial DNA, causing structural damage and inhibiting replication and repair, inactivating enzymes, disrupting catabolic processes, altering protein expression, and impairing the respiratory chain [35,36]. Additional studies suggest that silver ions affect the 30S ribosomal subunit, impairing protein synthesis and further suppressing ATP production [37]. All in all, these mechanisms weaken bacteria and their capacity for defense against antibiotics, facilitating antibiotic penetration, and causing cellular leakage, leading to cell death.

Accordingly, numerous studies have reported that combining antibiotics such as ampicillin, kanamycin, chloramphenicol, enoxacin, neomycin, and tetracycline, with silver ions and AgNPs, either conjugated or co-administered, can significantly enhance the antimicrobial activity of both compounds to treat bacteria, even when silver and antibiotics alone exhibited limited antimicrobial activity, as reviewed and discussed in Section 4. Fortunately, silver is not a threat to eukaryote cells that can manage large amounts of silver ions. Numerous investigations have consistently demonstrated that silver ions exhibit a high level of safety on mammalian cells and human health, providing reassuring results, and emphasizing their use as a reliable and well-tolerated antimicrobial option [38].

In this review, the mechanisms underlying the synergistic effects between antibiotics and AgNPs as reservoirs of silver ion release, and recent advancements in this field, are reviewed, integrating them with historical insights back to the use of colloidal silver since the mid-18th century. This approach, combining past and present knowledge, provides a comprehensive framework to guide future research and prevent the loss of valuable information [39,40].

## 2. Silver Colloids as Antibiotics: An Historical Perspective

**Figure 1:**
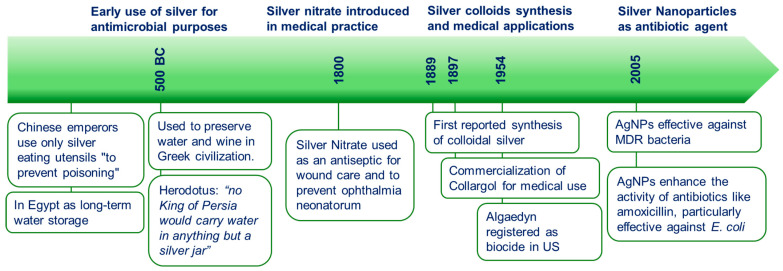
Historical timeline of silver ions in antimicrobial use and research.

Silver has a long history of medical use (Figure 1), dating back to ancient civilizations where it was valued for its antimicrobial properties. The earliest recorded application of silver for antimicrobial purposes can be traced to ancient Greece, where silver vessels were used to preserve drinking water and wine. While its antimicrobial effects were empirically recognized, the underlying mechanisms remained unknown at the time. In the 19th century, silver found its way into formal medical practice. Silver nitrate became a widely used antiseptic for wound care and was also employed to prevent ophthalmia neonatorum in newborns [41,42].

In 1889, M.C. Lea synthesized citrate-stabilized silver colloids, marking the first documented production of colloidal silver [43]. This formulation, which yielded AgNPs with a diameter of approximately 10 nm, demonstrated antimicrobial activity, paving the way for medical applications. By 1897, a colloidal silver product based on this, “Collargol” began to be commercialized for medical use [44]. Mid-20th-century advancements included the development of gelatin-stabilized disinfectant silver colloids, measuring 2–20 nm [45], and other silver colloid-based disinfectants [46,47]. Other colloidal silver products similar to Collargol, such as Argyrol and Protargol, became widely popular as over-the-counter treatments for conditions such as syphilis and bacterial infections over a period of 50 years. In addition to medical applications, colloidal silver has been also extensively used as a biocide. The earliest nanosilver product registered under the U.S. Federal Insecticide, Fungicide, and Rodenticide Act was Algaedyn in 1954 [48]. These and other products products included nanoparticles with sizes ranging from 2 to 20 nm along with silver ions and bulk particles.

Despite this progress, the use of silver in medicine declined in the 20th century with the advent of antibiotics, which provided a more targeted approach to treating infections. However, the increasing prevalence of antibiotic-resistant pathogens in recent decades has revived the interest in silver as an alternative or adjunct to conventional antibiotics. The development of nanotechnology in the late 20th century led to a renewed focus on silver nanoparticles (AgNPs). Different studies shed more light on the potential of AgNPs as effective antimicrobial agents against a wide range of pathogens, including antibiotic-resistant strains. A major milestone in this area was the demonstration that AgNPs can be used to combat MDR bacteria, a growing concern in healthcare [19,49].

Recent research has focused on optimizing the synthesis of AgNPs to enhance their antibacterial activity while reducing potential cytotoxicity, and new methods have been developed to tailor their size, allowing for controlled silver ion release [20]. Additionally, the synergistic effects of AgNPs combined with other antibiotics have been explored to improve treatment outcomes and address bacterial resistance [50]. This combination approach has proven particularly effective against Gram-negative bacteria, which are often more resistant to antibiotics due to their protective cell walls. These advancements highlight the growing potential of AgNPs as a valuable adjunct in combating antibiotic-resistant infections and their versatility in modern antimicrobial therapies. This ability of AgNPs has led to their integration into different medical devices and consumer products, such as wound dressings, catheters, and coatings for implants, helping prevent infections and enhancing the effectiveness of traditional antibiotics [51].

## 3. Mechanisms of Action and AgNPs Properties Influencing Antimicrobial Synergy

### 3.1. Mechanisms of Action of AgNPs and AgNPs-Based Antibiotic Therapies

**Figure 2:**
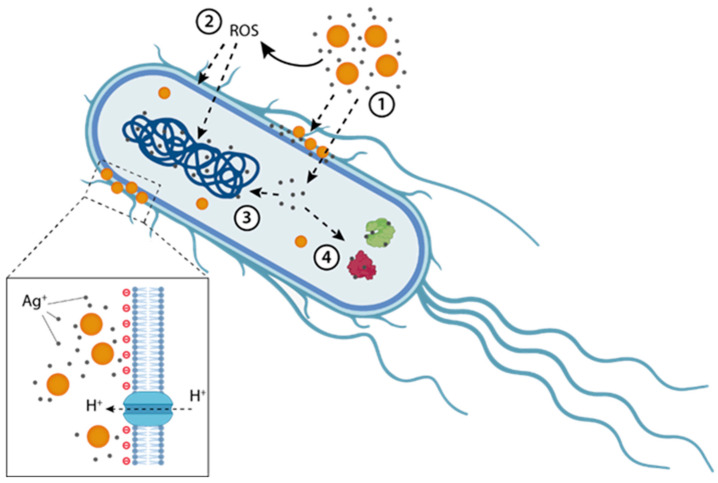
Antibacterial mechanisms of action. Proposed mechanisms of AgNP-related bacterial damage. (1) Silver ions release is promoted by acidic and aerobic environment. (2) Formation of ROS, which then damage both the membrane lipids and DNA. (3–4) Ag^+^ uptake can be promoted by membrane damage (although they might enter also through membrane channels). Ag^+^ ions may bind intracellular proteins and the bacterial chromosome, upon entering the cytosol, thus influencing metabolic activity and replication. (Inset) positively charged AgNPs may be attracted by negatively charged bacterial membranes, leading to higher local doses of NPs. Here, the proton motive force takes place, causing a local decrease in pH. This can further promote the dissolution of AgNPs, resulting in a local higher Ag^+^ concentration. In this picture, a Gram-negative bacterium has been taken as the model microorganism. Adapted from Sklodowski et al. [56] © 2023 by the authors. Licensee MDPI, Basel, Switzerland, an open access article distributed under the terms and conditions of the Creative Commons Attribution (CC BY) license.

As discussed in the previous section, silver has been used as an antimicrobial agent since ancient times. However, it is only in recent decades that the mechanisms underlying its antimicrobial activity have started to be elucidated, although a complete understanding remains elusive [34,52,53]. Nonetheless, three primary pathways can be identified (Figure 2). The first pathway involves direct interaction of AgNPs and silver ions with the bacterial membrane, leading to alterations in membrane permeability, structural damage, and eventual rupture, which causes leakage of cellular (cytoplasmatic) contents. The second pathway comes from the internalization of AgNPs and silver ions into bacteria, where they destabilize vital biomolecules, including proteins, lipids, and DNA. This disruption impedes essential cellular functions, such as protein synthesis, respiratory chain function, ATP production, gene transcription, and DNA replication. The third pathway is the oxidation and posterior corrosion and transformation of AgNPs into silver ions in biological environments, which disrupts bacterial cellular processes, including disulfide bond formation, metabolism, and iron homeostasis. These disruptions lead to an increase in reactive oxygen species (ROS) production, which, in turn, enhances bacterial membrane permeability [17,26,54,55].

These pathways often operate simultaneously and are interconnected, collectively weakening bacterial metabolism and overwhelming defenses. This enhances antibiotic effectiveness, increases bactericidal efficacy, and allows for lower antibiotic dosages, thereby reducing potential toxicity to human cells [21,26]. Even more, silver activity not only can boost the effectiveness of a broad range of antibiotics against bacteria (Table 1) in different metabolic states but also can restore susceptibility to antibiotics in resistant bacterial strains [20]. Another advantage of conjugating AgNPs to antibiotics is that their pharmacokinetic profiles become unified, altering their biodistribution and ensuring accumulation at the site of infection [57,58]. Furthermore, NPs may offer protection to the antibiotic molecules, shielding them from degradation before reaching their target.

Finally, AgNPs can be used in combination with antibiotics and other treatments, such as hyperthermia. AgNPs are excellent local conductors of heat, making them effective at transmitting heat to adjacent tissues. For instance, when combined with hyperthermia, this can result in bacterial biofilm death [57]. Here, it is important to note that AgNPs alone are not sufficient to treat biofilms due to aggregation issues in complex biological media that reduce their bactericidal efficacy [58]. To address this, AgNPs can be modified with antibiotics such as amikacin (AK) and combined with hyperthermia, creating a multimodal strategy to combat antibiotic-resistant bacteria in biofilms [26,57].

**Figure 3:**
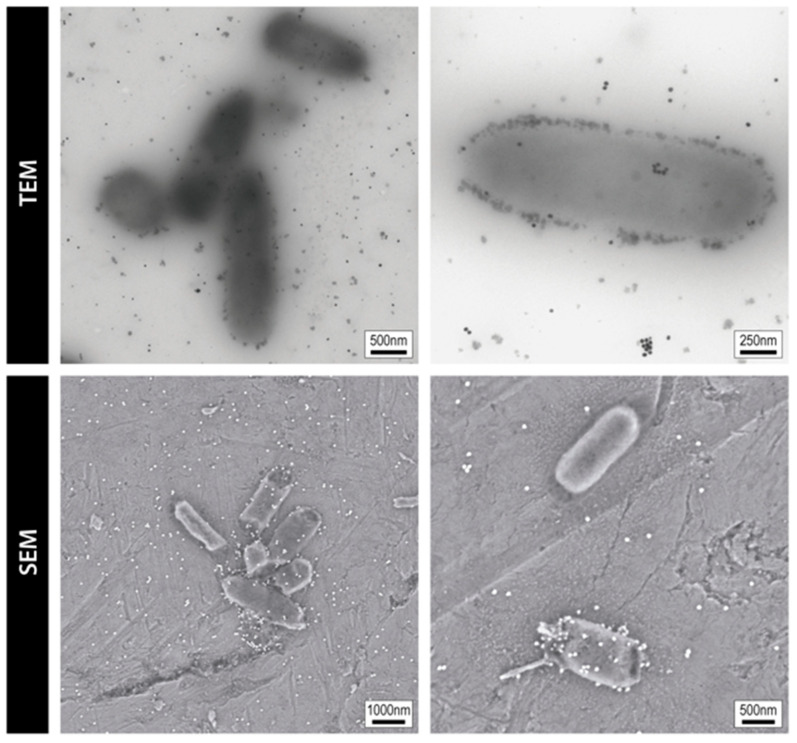
Ag–bacteria interaction. Bright-field and dark-field transmission and scanning electron microscopy images of *E. coli* inoculums incubated overnight with 20 nm AgNPs. In the TEM images, particles can be seen surrounding the bacterium (at the membrane), and some appear to overlap with the bacterial area, but TEM itself cannot confirm whether the NPs are located inside or on/under the surface. In contrast, in the SEM images, the particles visible due to contrast differences are located only on the external surface. Scale bars in TEM images are 500 nm (**left**) and 250 nm (**right**), and in SEM images are 1000 nm (**left**) and 500 nm (**right**).

The direct observation of the exposed bacteria to AgNPs by electron microscopy provides visual insights into the interaction between AgNPs and bacteria, offering a clearer understanding of the mechanistic aspects of their biocidal effects. For instance, Figure 3 shows TEM images revealing 20 nm AgNPs attached to the *E. coli* cell wall, with NPs distributed across the bacterial area. While some NPs appear in close proximity to bacteria, it remains unclear whether they are internalized or simply adhered to the outer membrane. In contrast, SEM offers higher-resolution imaging of bacterial morphology, showing both intact cells and others exhibiting a shrunken, near-death appearance (data not published).

Beyond antibacterial assays and phenotypic observations, transcriptomic and proteomic studies have recently provided deeper insights into the molecular responses of bacteria to AgNP exposure. For instance, global transcriptional profiling of *Escherichia. coli* and *Pseudomonas aeruginosa* has revealed the upregulation of oxidative stress response genes (e.g., *soxS*, *katG*, *oxyR*) and metal efflux systems, alongside the downregulation of genes involved in central metabolic pathways, membrane transport, and protein synthesis [59]. Proteomic analyses have confirmed these findings, showing reduced expression of ribosomal proteins and enzymes essential for glycolysis, the TCA cycle, and amino acid biosynthesis upon uptake of silver ions into the cell [27,60]. These molecular disruptions also impair bacterial energy production, redox balance, and replication, contributing to growth inhibition and enhanced sensitivity to antibiotics. Such systems-level data offer compelling evidence of how AgNPs reprogram bacterial metabolism and defense responses, further validating their role in overcoming multidrug resistance.

### 3.2. Influence of AgNP Physicochemical Properties on Ion Release and Antimicrobial Synergy

Building on the previous mechanisms, it is clear that the physicochemical characteristics of AgNPs (such as size, shape, and surface properties) play a crucial role in determining their antimicrobial performance and synergy with the three main antimicrobial pathways described earlier. These parameters govern the dissolution kinetics of AgNPs into bioactive silver ions (Ag^+^), modulating interactions with bacterial cells, and affecting the local concentration of ions at the infection site, which in turn enhances antibiotic activity.

Regarding size, the smaller the AgNPs, the higher the surface area-to-volume ratio, which facilitates faster oxidation and dissolution in biological environments, leading to more rapid and abundant Ag^+^ ion release [61,62]. This ion release is central to their antimicrobial activity, as the solubility and reactivity of NPs are governed by surface atom coordination, which in turn depends on curvature and particle size [57,58]. By tailoring NPs size, it is possible to control the dissolution rate and optimize the antibacterial response. Accordingly, smaller AgNPs exhibit consistently higher dissolution rates, translating into stronger bactericidal efficacy. For example, Shaverdi et al. reported that AgNPs around 5 nm exhibited superior activity against oral bacteria, achieving minimum inhibitory concentrations (MICs) as low as 25–50 µg/mL [63]. Similar trends have been observed for *E. coli*, *P. aeruginosa*, and *Staphylococcus aureus* [17,38].

Importantly, when Ag is combined with antibiotics, the localized delivery of Ag ions becomes crucial, as Ag ions in biological media are short-lived due to their tendency to form insoluble compounds, such as AgCl. Hence, it is by targeting Ag ion release directly to the bacterial cell wall, where antibiotics are active, that it offers the possibility to maximize their effect. Thus, smaller AgNPs (≤10 nm) also consistently show enhanced synergy with antibiotics. Several studies have confirmed that these particles outperform their larger counterparts in combination therapies. Agnihotri et al. [64], for instance, observed significant MIC reductions when combining sub-10 nm AgNPs with antibiotics such as kanamycin and tetracycline (Agnihotri 2014).

Regarding shape, NP geometry influences the proportion of reactive crystalline facets. Nanoparticles with triangular, prismatic, or cubic morphologies expose different crystallographic facets (e.g., {111}, {100}) that vary in surface energy and, consequently, dissolution behavior. Triangular platelets, in particular, exhibit enhanced Ag^+^ ion release and greater interaction with bacterial membranes due to their high-energy facets [65].

Regarding surface characteristics, it is known that the zeta potential of AgNPs affects their electrostatic interaction with negatively charged bacterial membranes. Positively charged or slightly negative AgNPs are more likely to adhere to bacterial surfaces, enhancing local Ag^+^ concentration and facilitating membrane disruption. This also aids in the intracellular accumulation of antibiotics. For instance, Shahverdi et al. [63] demonstrated that AgNPs with appropriate surface charges substantially increased the inhibition zone of otherwise ineffective antibiotics like vancomycin and penicillin G against resistant strains.

Surface functionalization is another key parameter. Stabilizing agents and surface coatings such as citrate, polyethylene glycol (PEG), or antibiotics themselves can influence the colloidal stability, aggregation behavior, and dissolution kinetics of AgNPs. For example, Deng et al. [17] showed that tetracycline-conjugated AgNPs exhibited increased Ag^+^ ion release and stronger antibacterial effects due to enhanced complex formation. We have recently reported that AgNPs functionalized with amikacin and polyethylene glycol exhibited 10-fold increased efficacy against MDR biofilms compared to the antibiotic alone [26].

The aggregation state of AgNPs in biological fluids affects their performance. Aggregated particles have a reduced surface area exposed to the medium, resulting in lower dissolution rates and diminished antimicrobial activity. This effect is particularly relevant in physiological conditions or within biofilms, where proteins, salts, and other biomolecules may induce nanoparticle clustering.

Lastly, depending on the synthesis method and intended application, AgNPs-based antibiotics combined with antibiotics can be presented as colloids, suspensions, and gels, or incorporated into various products, such as medical dressings. All of them vary in terms of the physicochemical properties described above. It is important to note that these differences in properties can lead to distinct antibacterial mechanisms and diverse bacterial responses [66,67].

Importantly, these properties are not only individually important or dependent on the synthetic method and surface coating [68], which may modify ion release behavior and biological interactions. They also depend on the exposure medium and are interdependent with one another. For instance, the exposure media can modify the surface charge of the NPs, which in turn may influence aggregation, modifying the size of the particles and impacting dissolution kinetics [69]. A summary of their influence on Ag^+^ ion release and antibiotic synergy is presented in Table 2.

## 4. Advances in the Combined Use of Silver and Antibiotics

### 4.1. Overview of Early Research on Silver–Antibiotic Synergy

The potential of Ag ions and AgNPs to enhance the activity of common antibiotics has been investigated for over two decades. The earliest study we identified was conducted by Li et al., employing polydisperse AgNPs (c.a. 20 nm in diameter) with cubic shape and amoxicillin (β-lactam antibiotic) [49]. In this study, the MIC in *E. coli* for amoxicillin was found to be 0.525 mg/mL and 40 µg/mL for AgNPs. When co-incubated, 0.150 mg/mL amoxicillin and 5 µg/mL AgNPs demonstrated the same antibacterial efficiency as much higher doses of either compound alone. Furthermore, the combination significantly delayed the exponential and stationary phases. The study also assessed the impact of pre-incubating bacteria with AgNPs, and potential mechanisms of action were proposed to explain these observations.

Following this study, researchers began investigating similar strategies in this field. For instance, Shahverdi et al. examined the antibacterial activity of 14 different antibiotics (penicillin G, amoxicillin, carbenicillin, cephalexin, cefixime, gentamicin, amikacin, erythromycin, tetracycline, cotrimoxazole, clindamycin, nitrofurantoin, nalidixic acid, and vancomycin) using the disk diffusion method in the presence of polydisperse AgNPs (ranging from 5 to 32 nm) against *E. coli* and *Staphylococcus. Aureus* [63]. The presence of AgNPs led to an increased zone of inhibition for penicillin G, amoxicillin, erythromycin, clindamycin, and vancomycin, with the most significant increases observed for vancomycin, amoxicillin, and penicillin G. Among the results, notably, penicillin G alone had no inhibitory effect on *Staphylococcus. aureus*, but in combination with AgNPs, an inhibition zone of 12 mm was observed. Similarly, vancomycin alone was ineffective against *Staphylococcus. aureus*, whereas with AgNPs, it produced a 13 mm inhibition zone. The amoxicillin–AgNPs combination demonstrated the greatest increase, with the inhibition zone expanding from 7.5 mm (amoxicillin alone) to 14.0 mm.

Fayaz et al. evaluated the enhancement of antimicrobial activity by combining AgNPs (ranging from 5 to 40 nm) with antibiotics such as ampicillin, kanamycin, erythromycin, and chloramphenicol [70]. Their study assessed the effects against both Gram-positive (*S.aureus*, *Bacillus subtilis*) and Gram-negative bacteria (*E. coli*, *P. aeruginosa*). Among the tested combinations, ampicillin exhibited the strongest synergistic effect, showing the greatest enhancement in antibacterial activity against the studied strains. Gosh et al. employing AgNPs of varying morphologies demonstrated increased antibacterial effects when combined with beta-lactam (piperacillin) and macrolide (erythromycin) antibiotics [71]. Specifically, the authors found a 3.6-fold and 3-fold enhancement, respectively, in activity against multidrug-resistant *Acinetobacter baumannii*. Furthermore, synergistic effects were observed between AgNPs and chloramphenicol or vancomycin against *P. aeruginosa*.

These early research efforts were also dedicated to gaining in-depth mechanistic insights. One of the first comprehensive studies was conducted by Morones et al. [21]. This study investigated the ability of Ag^+^ ions to enhance antibiotic activity against model Gram-negative bacteria (*E. coli*) by analyzing its effects on bacterial cellular processes. Their findings revealed that silver disrupts disulfide bond formation, metabolism, and iron homeostasis, leading to increased ROS production and membrane permeability, thereby enhancing the efficacy of three major antibiotic classes against *E. coli*: ß-lactams (ampicillin), which target cell wall synthesis; quinolones (ofloxacin), which inhibit DNA replication and repair; and aminoglycosides (gentamicin), which bind to ribosomes and cause protein mistranslation. In addition, Ag^+^ ions were shown to restore antibiotic susceptibility in resistant bacterial strains and to sensitize Gram-negative bacteria to vancomycin, a Gram-positive-specific antibiotic, in both urinary tract infection and peritonitis mouse models. Furthermore, silver–antibiotic combinations effectively eradicated bacterial persistent cells and enhanced antibacterial activity against biofilm-associated infections, as demonstrated in vitro and in a mouse biofilm infection model.

Herisse et al. expanded those previous findings by systematically evaluating silver’s impact on both bactericidal and bacteriostatic antibiotics [72]. This study demonstrated that Ag^+^ ions enhanced aminoglycosides efficacy. In these cases, silver enhanced the activity of the aminoglycosides mainly by bypassing the proton motive force at the protein translation level, while oxidative stress or Fe–S cluster destabilization were not essential factors for the potentiating effect of silver. The research also showed that silver enabled aminoglycosides to effectively target gentamicin-resistant *E. coli* mutants (K12) and the resistant anaerobic pathogen *Clostridium difficile*. In detail, according to changes in MIC values, silver was found to be most potent with the aminoglycosides gentamicin, kanamycin, tobramycin, and streptomycin, as MIC value decreased by more than 10-fold. Moreover, they reported a slight potentiating effect (less than 20%) when silver was used in conjunction with quinolone (nalidixic acid and norfloxacin) or with chloramphenicol.

### 4.2. Recent Advances in Silver and Antibiotic Combinations (2015–2025)

Recently, novel approaches and discoveries have led to a deeper exploration of the mechanisms underlying this synergy and its potential applications in combating antibiotic resistance. For instance, Deng et al. showed that the incorporation of AgNPs into antibiotic formulations allows for a reduction in the minimum administrable dose of the antibiotic, potentially leading to decreased side effects and improved patient tolerance [17]. In detail, the study investigated the synergistic effect of AgNPs with vaampicillin, penicillin, enoxacin, kanamycin, neomycin, and tetracycline against multidrug-resistant *Salmonella* Typhimurium. The results showed that enoxacin, kanamycin, neomycin, and tetracycline enhanced bacterial growth inhibition when combined with AgNPs, while ampicillin and penicillin did not, concluding that the tetracycline–AgNPs complex enhances Ag^+^ release, creating a high local concentration of Ag^+^ that contributes to bacterial growth inhibition.

Similarly, Malaong et al. explored the synergistic effect of combining AgNPs with ceftazidime, imipenem, meropenem (beta-lactam), and gentamicin (aminoglycoside) against *Burkholderia pseudomallei*, the cause of melioidosis [55]. The highest enhancement was observed with gentamicin. Scanning electron microscopy showed severe bacterial damage, confirming the combination’s effectiveness in overcoming resistance. Khleifat et al. evaluated the antibacterial activity of AgNPs synthesized using *Aspergillus flavus* filtrate showed broad-spectrum antibacterial activity against *S.aureus*, *P. aeruginosa*, *Enterobacter cloacae*, *E. coli*, and *Shigella* sp, with the strongest effect against *E. coli* and the weakest against *P. aeruginosa* [73]. When combined with antibiotics (ampicillin, erythromycin, ceftriaxone, vancomycin, azlocillin, amoxicillin, clindamycin, aztreonam, ciprofloxacin), AgNPs enhanced antimicrobial efficacy by up to 31-fold against these bacteria.

Other recent studies also show that AgNPs could serve as potential standalone antibiotic therapies or antibiotic adjuvants. For instance, Dove et al. examined the antimicrobial efficacy of different AgNPs against *E. coli*, *P. aeruginosa*, *A. baumannii*, and *S.aureus* (methicillin-resistant and susceptible strains) [24]. The most effective AgNP formulation exhibited MICs lower than 10 μg/mL against multidrug-resistant Gram-negative bacteria. Synergy was observed when AgNPs were combined with aminoglycosides, reducing MICs by approximately 22-fold. A study on *A. baumannii* isolates from Riyadh found that 8.19% were pan-drug resistant, including resistance to colistin and imipenem. Combining AgNPs with these antibiotics significantly lowered their MICs, with colistin-AgNP combinations showing the strongest synergy. Most isolates were from respiratory infections, and colistin resistance varied by sex [74].

### 4.3. Restoring Antibiotic Susceptibility

Recently, it has been also shown that in certain instances, the presence of silver appeared to restore antibiotic susceptibility to antibiotic-resistant bacterial strains, as exemplified in the case of *mcr-1*–positive *E. coli* exposed to colistin and AgNPs [75]. In this study, using X-ray crystallography, it was found that Ag^+^ inhibits the MCR-1 enzyme by replacing Zn^2+^ in its active site, forming a tetra-silver center that blocks substrate binding. Additionally, Ag+ slows resistance development and, when combined with colistin at low concentrations, effectively reduced bacterial load and dermonecrotic lesions in infected mice.

### 4.4. Antifungal Effects

Leonhard et al. expanded the combination of AgNPs with antibiotics for antibacterial and antifungal effects [76]. This study optimized the conjugation of amphotericin B (AmB) to AgNPs and characterized their physicochemical properties, cytotoxicity, and biological activity. The optimal Ag/AmB molar ratio of 1:1 produced nanoconjugates of ~170 nm and achieved high drug-loading efficiency (higher than that of micellar and liposomal formulations). The AgNP-AmB retained both silver’s bactericidal and AmB’s antifungal effects, while their spontaneous association with plasma lipoproteins reduced cytotoxicity to red blood cells with minimal impact on antibacterial activity. Notably, the antifungal efficacy was maintained or enhanced, highlighting the potential of AgNP-AmB as an improved antifungal nanocarrier.

### 4.5. Biofilm Eradication

The combination of AgNPs and antibiotics has proven efficacious not only in solution but also against persistent bacterial biofilms [26]. This study synthesized highly monodispersed AgNPs (17 nm) functionalized with mercaptopoly(ethylene glycol) carboxylic acid (mPEG-COOH) and amikacin (AK) (AgNPs-mPEG-AK) and evaluated their antibacterial efficacy. Characterization was performed using spectroscopy and microscopy, and susceptibility was tested against 12 multidrug-resistant (MDR)/extensively drug-resistant (XDR) *A. baumannii*, *E. coli*, *Klebsiella pneumoniae*, and *P. aeruginosa* strains. AgNPs-mPEG-AK was 10 times more effective than AK alone, showing bactericidal effects in all tested strains within 48 h. When combined with hyperthermia (1–3 pulses at 41–42 °C for 15 min), it eradicated 75% of planktonic strains and significantly reduced *P. aeruginosa* biofilm formation.

Similarly, a study evaluating the antibacterial and antibiofilm effects of Curcuma aromatica-derived AgNPs (CAAgNPs) against multidrug-resistant *P. aeruginosa* (NCIM 5029, PAW1) and *S.aureus* (NCIM 5021, S8) showed effective inhibition of biofilm formation and bacterial growth, with MICs and MBCs ranging from 8 to 128 μg/mL. The nanocomposites also enhanced antibiotic activity, particularly against the biofilm forms of these pathogens, demonstrating potential for use in wound care and medical devices [77]. Another study combining AgNPs and kanamycin, coated with polydopamine to develop pH-responsive nanocomposites found that the nanocomposites responded to the acidic biofilm environment, triggering on-demand drug release and dispersing biofilms. The nanocomposites demonstrated enhanced antimicrobial activity against *S.aureus*, *Streptococcus pneumoniae*, *P. aeruginosa*, and *E. coli BL21* [78].

In this regard, AgNPs-antibiotic conjugates can be highly effective in managing wound infections by incorporating the conjugates into dressings or topical formulations, ensuring sustained release of both AgNPs and antibiotics to target bacteria and promote healing. For instance, Wali et al. investigated the antibacterial effect of colistin combined with AgNPs and decellularized human amniotic membrane (HAM) as a dressing for burn wounds infected with multidrug-resistant bacteria [79]. The combination of colistin and AgNPs in HAM showed enhanced antimicrobial activity, faster wound healing, and increased collagen deposition. The treatment significantly reduced bacterial load, with *P. aeruginosa* and *K. pneumoniae* being the most common pathogens.

Additionally, this approach has shown efficacy in proof-of-concept studies preventing implant-related infections, like those associated with orthopedic implants or catheters, by coating the implant surfaces with AgNPs and antibiotics to prevent biofilm formation [80]. Moreover, silver–antibiotic combinations can be effective against respiratory, urinary, and bloodstream infections caused by MDR bacteria [81]. Overall, in clinical practice, the use of AgNPs in combination with antibiotics holds promise for broadening antimicrobial effectiveness, enhancing the efficacy of existing antibiotics, and overcoming resistance mechanisms. A summary of the studies analyzed in this review is provided in Table 3.

## 5. Conclusions and Future Perspectives

The combination of AgNPs with antibiotics presents a promising strategy for combating bacterial infections, particularly those caused by antibiotic-resistant strains. Various mechanisms have been proposed and observed for the enhancement of antibiotic efficacy by AgNPs, including bacterial membrane disruption, interference with essential cellular processes, DNA damage, and ROS overproduction. These mechanisms place a significant energetic burden on bacterial defenses, leading to improved bactericidal activity, lower required antibiotic doses, and potentially reducing the emergence of resistance. Furthermore, the ability to modulate Ag^+^ ion release via controlled AgNP oxidation and dissolution (which depends on factors such as nanoparticle size, the medium used, and the reactivity of the particles, all of which can influence how the bacteria respond to the antibiotic) can add precision to antimicrobial applications, making AgNPs a valuable complement to existing and future antibiotic therapies.

This review has provided an overview of the mechanisms of action and recent advancements in the synergistic effects of AgNP-antibiotic combinations. Through this analysis, three critical challenges to fully realize their clinical potential have been identified:

1. The potential for resistance development against silver. While AgNPs exhibit broad-spectrum antimicrobial activity, prolonged exposure to sub-lethal concentrations may lead to bacterial adaptation and silver resistance. Studies have shown that bacteria exposed to AgNPs can develop increased tolerance, potentially affecting the efficacy of both silver-based and conventional antibiotic treatments. Mechanistically, silver resistance has been linked to genetic determinants such as the *sil* operon, which encodes proteins involved in silver ion efflux (*SilP*, *SilCBA*) and periplasmic sequestration (*SilE*), thereby reducing intracellular Ag^+^ accumulation [82,83]. Additionally, endogenous efflux systems like *SilCFBA*, as well as *CusCFBA* (originally characterized for copper), have been implicated in silver detoxification [82,84].
  Biofilm-mediated tolerance also contributes to resistance by limiting the penetration of AgNPs and silver ions through the extracellular polymeric matrix and mitigating oxidative stress via localized redox buffering. Furthermore, horizontal gene transfer and co-selection with antibiotic resistance genes may accelerate the spread of silver resistance, especially in clinical and environmental settings. These findings underscore the need for prudent AgNP use and the development of dosing strategies that minimize resistance development while preserving therapeutic efficacy. To mitigate all these risks, future research should focus on optimizing dosing strategies, elucidating resistance mechanisms at the molecular level, and designing AgNP formulations that minimize resistance selection while maximizing synergy with antibiotics.
2. Scalable Production. As with many other nanomaterials, the clinical translation of AgNP-antibiotic therapies requires regulatory approval, which demands adherence to Good Manufacturing Practice (GMP) standards. Challenges such as ensuring batch consistency, characterizing physicochemical properties, and sourcing GMP-compliant reagents must be addressed. Streamlining production protocols and establishing standardized evaluation frameworks will be crucial for advancing AgNP-based therapeutics to clinical application.
3. Regulatory approval. One significant limitation for the clinical development of AgNP-based therapies is the current lack of harmonized regulatory frameworks and well-defined cytotoxicity thresholds for human applications. Despite increasing evidence of AgNPs’ antimicrobial potential, their physicochemical variability leads to inconsistent toxicity profiles, complicating risk assessment and regulatory approval. Although different studies suggest that concentrations below 10 µg/mL are safe for human cells, the variability in formulations and testing approaches remains a significant challenge in establishing universal safety limits. Moreover, stabilizing agents used to ensure colloidal stability may themselves contribute to cytotoxicity or immunogenicity. To address these challenges, it is essential to implement standardized evaluation protocols for AgNP formulations, including in vitro and in vivo toxicity screening under conditions that mimic physiological environments. Establishing universal parameters, such as maximum tolerable dose, exposure limits, and biocompatibility standards, would enable more consistent safety assessments. Surface engineering approaches that use biocompatible, FDA-approved coatings (e.g., PEG, albumin, or phospholipid layers) can reduce toxicity while maintaining stability and efficacy.
4. Long-Term Toxicity, Organ Accumulation. Beyond acute cytotoxicity, the long-term safety profile of NPs, including AgNPs, remains an area of concern. Multiple studies have shown that systemically administered NPs accumulate mainly in the liver and spleen, and much less in kidneys and lungs, with retention times influenced by NP size, surface coating, and administration route. Persistent exposure may lead to oxidative stress, inflammatory responses, or subtle immunomodulatory effects, particularly with non-degradable coatings. Although some formulations appear to be well tolerated in animal models, comprehensive long-term studies are still lacking.
5. Variability in reported synergistic effects. Factors such as nanoparticle size, surface chemistry, and the choice of antibiotic play a critical role in determining synergistic effects. Optimizing these parameters will not only enable the development of tailored AgNP formulations but also enhance the interpretation of findings, contributing to the advancement of the field.

In the coming decade, AgNP-antibiotic combinations may revolutionize infection treatment, particularly for life-threatening conditions such as sepsis and multidrug-resistant bacterial infections. Additionally, AgNPs could be integrated into medical devices and hospital surfaces to prevent nosocomial infections. However, practical considerations, including cost-effectiveness, large-scale production feasibility, and formulation stability, must be addressed to enable widespread clinical adoption.

## Figures and Tables

**Table 1:** Classification of antibiotics: types, mechanisms, and bacterial sensitivity.

Type	Members	Characteristics	Mode of Action	Spectrum
β-Lactams	Penicillins, Cephalosporins, Carbapenems, Monobactams	β-lactam ring	Inhibits cell wall synthesis (peptidoglycan)	Gram-positive, some Gram-negative
Aminoglycosides	Kanamycin, Streptomycin, Gentamicin, Neomycin, Amikacin, Netilmicin, Tobramycin	Amino sugars linked together	Inhibits protein synthesis (30S ribosomal subunit)	Gram-negative
Tetracyclines	Tetracycline, Oxytetracycline, Chlortetracycline, Doxycycline, Minocycline	Naphthalene tetracyclic structure (four rings)	Inhibits protein synthesis (30S ribosomal subunit)	Gram-positive and Gram-negative
Macrolides	Erythromycin, Oleandomycin, Azithromycin, Clarithromycin, Josamycin, Telithromycin	Large macrolactone rings with aminated sugars	Inhibits protein synthesis (50S ribosomal subunit)	Gram-negative
Chloramphenicol	—	Synthesized in the laboratory	Inhibits protein synthesis (50S ribosomal subunit)	Gram-positive and Gram-negative
Rifamycins	Rifampin, Rifabutin	—	Inhibits RNA synthesis (RNA polymerase)	Gram-positive and Gram-bacteria, Tuberculosis
Sulfonamides	Sulfacetamide, Silver sulfadiazine	Chemotherapeutic, synthesized in the laboratory	Inhibits PABA (folic acid) synthesis	Gram-positive and Gram-negative
Quinolones	Nalidixic acid, Ciprofloxacin, Moxifloxacin, Levofloxacin, Ofloxacin	Synthesized in the laboratory	Inhibits DNA replication	Gram-positive and Gram-negative
Polypeptides	Bacitracin, Colistin, Polymyxin B	—	Inhibits cell wall synthesis (peptidoglycan) and alters plasma membrane permeability	Gram-positive and Gram-negative

**Table 2:** Influence of AgNP physicochemical properties on silver ion release and antibiotic synergy.

Property	Effect on Ag^+^ Ion Release	Impact on Antimicrobial Synergy
Size	Smaller size increases surface area and dissolution rate	Enhances Ag^+^ availability; boosts antibiotic penetration and efficacy
Shape	Triangular/cubic shapes expose high-energy facets (e.g., {111})	Higher Ag^+^ release and membrane interaction; shape-dependent synergy
Surface charge	Positive or mildly negative charge enhances bacterial membrane binding	Increases local NP and Ag^+^ accumulation; facilitates antibiotic uptake
Surface coating	Functional groups (e.g., citrate, PEG) modulate ion release	Affects colloidal stability and pharmacokinetics of NP-antibiotic conjugates
Aggregation state	Aggregated NPs release fewer ions due to lower exposed surface	Reduced efficacy in biological fluids and biofilms

**Table 3:** Studies analyzed in this review on silver–antibiotic synergies, organized by antibiotic class.

Antibiotic Class	Antibiotic Combined with AgNPs	Bacterial Strains	Results	Author-Year
β-lactam	Amoxicillin	*E. coli*	AgNPs enhanced antibacterial activity; 0.150 mg/mL amoxicillin and 5 µg/mL AgNPs showed same effect as higher doses	Li et al. [49]
β-lactam, Macrolide	Piperacillin, Erythromycin, Chloramphenicol, Vancomycin	*A. baumannii*, *P. aeruginosa*	3.6-fold enhancement with piperacillin, 4.9-fold with chloramphenicol, 4.2-fold with vancomycin	Gosh et al. [71]
β-lactam, Macrolide	Penicillin G, Amoxicillin, Erythromycin, Clindamycin, Vancomycin	*E. coli*, *S.aureus*	Increased zone of inhibition, strongest with Vancomycin, Amoxicillin, Penicillin G	Shahverdi et al. [63]
β-lactam, Aminoglycoside	Ceftazidime, Imipenem, Meropenem, Gentamicin	*e*	Gentamicin showed highest enhancement	Malawong et al. [55]
β-lactam, Macrolide, Aminoglycoside	Ampicillin, Kanamycin, Erythromycin, Chloramphenicol	*S.aureus*, *B. subtilis*, *E. coli*, *P. aeruginosa*	Ampicillin showed the strongest synergistic effect	Fayaz et al. [70]
β-lactam, Quinolone, Aminoglycoside	Ampicillin, Ofloxacin, Gentamicin, Vancomycin	*E. coli*	Silver enhances ROS production, increases membrane permeability, and restores antibiotic efficacy	Morones et al. [21]
β-lactam, Tetracycline, Aminoglycoside	Ampicillin, Penicillin, Enoxacin, Kanamycin, Neomycin, Tetracycline	*Salmonella* Typhimurium	Tetracycline formed Ag complexes, enhanced Ag^+^ release	Deng et al. [17]
β-lactam, Aminoglycoside, Macrolide, Fluoroquinolone	Ampicillin, Erythromycin, Ceftriaxone, Vancomycin, Azlocillin, Amoxicillin, Clindamycin, Aztreonam, Ciprofloxacin	*S.aureus*, *P. aeruginosa*, *E. cloacae*, *E.e*, *Shigella* sp.	Up to 31-fold enhancement in antimicrobial efficacy	Khleifat et al. [73]
Aminoglycoside	Aminoglycosides	*E. coli*, *P. aeruginosa*, *A. baumannii*, *S.aureus* (methicillin-resistant)	22-fold reduction in MIC with AgNPs	Dove et al. [24]
Aminoglycoside	Amikacin	*A. baumannii*, *E.e. pneumoniae*, *P. aeruginosa*	10× more effective than amikacin alone	Palau et al. [26]
Aminoglycoside	Kanamycin	*S.aureus*, *S. pneumoniae*, *P. aeruginosa*, *E. coli* BL21	Enhanced antimicrobial activity, on-demand drug release	Li et al. [78]
Aminoglycoside, Tetracycline	Gentamicin, Kanamycin, Tobramycin, Streptomycin, Spectinomycin, Tetracycline	*E. coli* K12, *C. difficile*	Silver enhanced efficacy by 10-fold or more for aminoglycosides	Herisse et al. [72]
Polymyxin	Colistin	*E. coli* (*mcr-1* positive)	AgNPs restored susceptibility to colistin	Zhang et al. [75]
Polymyxin	Colistin	Orthopedic implants or catheters	Prevention of implant-related infections and biofilm formation	Kadirvelu et al. [80]
Polymyxin	Colistin	*P. aeruginosa*, *K. pneumoniae*	Enhanced antimicrobial activity, faster wound healing	Wali et al. [79]
	Curcuma aromatica-derived AgNPs	*P. aeruginosa*, *S.aureus*	Inhibited biofilm formation and bacterial growth	Tawre et al. [77]
	Various antibiotics	Multidrug-resistant bacteria	broad antimicrobial activity	Feizi et al. [81]

## Data Availability

No new data were created or analyzed in this study.
